# A New Species of *Sinospelaeobdella* from China: *Sinospelaeobdella jiangxiensis* sp. n. (*Hirudinda, Arhynchobdellida, Haemadipsidae*)

**DOI:** 10.3390/ani15081079

**Published:** 2025-04-08

**Authors:** Tianyi Li, Yuhang Liu, Chen Zhang, Hao Gu, Zheng Cheng, Jie Peng, Jiang Feng, Ying Liu

**Affiliations:** 1 Jilin Provincial Key Laboratory of Animal Resource Conservation and Utilization, Northeast Normal University, Changchun 130117, China; lity734@nenu.edu.cn (T.L.); fengj@nenu.edu.cn (J.F.); 2 Jilin Provincial International Cooperation Key Laboratory for Biological Control of Agricultural Pests, Northeast Normal University, Changchun 130117, China; 3 Changchun Veterinary Research Institute, Chinese Academy of Agricultural Sciences, Changchun 130122, China; 4 Key Laboratory of Vegetation Ecology, School of Environment, Institute of Grassland Science, Northeast Normal University, Ministry of Education, Changchun 130117, China; 5 School of Life Science, Jilin Agricultural University, Changchun 130118, China

**Keywords:** Haemadipsidae, *Sinospelaeobdella*, DNA taxonomy, morphology, new species

## Abstract

We describe a new species (*Sinospelaeobdella jiangxiensis* **sp. n.**) from a natural cave in southwestern Jiangxi Province, China, according to external and internal morphology and COI gene sequences. *S. jiangxiensis* **sp. n.** is distinguishable from other species of *Sinospelaeobdella* primarily by morphological characteristics, including body length, the position of the fifth pair of eyes and the first pair of testes, the number of caeca, and the shape and number of auricular projections. We also estimate its phylogenetic position within the genus *Sinospelaeobdella.* Molecular phylogenetic analyses supported the morphological delimitation of *S. jiangxiensis* **sp. n.** as separate from *S. wulingensis.* Both the morphological and molecular data support *S. jiangxiensis* **sp. n.** as a new species, so the results provide critical data for the determination and conservation of *Sinospelaeobdella*, and enhance the documented diversity of this recently established genus.

## Simple Summary

Through integrative morphological and molecular phylogenetic analyses, the present study describes a novel species of *Sinospelaeobdella* which was established in 2019 and currently comprises only two species, enhancing the documented diversity of the new genus.

## 1. Introduction

Land leeches (Arhynchobdellida, Haemadipsidae) are blood-feeding annelids that live in forests and grasslands in tropical and subtropical regions. Over 70 species have been described at present. Based on the number of jaws, the terrestrial leeches can be classified into two clades: the duognathous clade (2-jawed), comprising 12 genera, and the trignathous clade (3-jawed), comprising 5 genera [1,2]. Recently, with continuous advancements in molecular research methodologies, significant revisions have been made to the classification of Haemadipsidae. Particularly, the 17 genera of the duognathous clade have been proposed for merger into a single genus *Chtonobdella*, while the three genera *Xerobdella*, *Mesobdella* and *Diestecostoma* in the trignathous clade were reclassified into the newly established family Xerobdellidae. Only the genera *Haemadipsa* and *Tritetrabdella* remain within Haemadipsidae [1,2,3].

*Sinospelaeobdella*, recently established in 2019, represents a novel genus within the trignathous clade. *Leiobdella,* in the duognathous clade, and *Sinospelaeobdella* are the only two genera of the family Haemadipsidae known to live in caves [4,5]. Only two species have been recorded currently in genus *Sinospelaeobdella*: *Sinospelaeobdella cavatuses* Yang 2009 and *Sinospelaeobdella wulingensis* Liu 2019. *S. wulingensis* is the type species of *Sinospelaeobdella* and was recently discovered in Hunan, Sichuan, and Guizhou Provinces [4,6,7]; *S. cavatuses* was discovered in Yunnan Province, China and Luang Namtha, Laos [2,8]. These two species of *Sinospelaeobdella* were both observed only in the humid karstic caves in southern China where small organisms are difficult to be found. Therefore, the potential species diversity of the genus needed to be discovered. The known species with several adaptive features, such as loss of body pigmentation, enlarged body papillae, giant suckers, and changes in feeding habits, have evolved into troglobites. They are monoecious, allogeneic, and have multiple pairs of caeca. Based on iDNA analyses of *S. wulingensis*, the species has been identified as mainly feeding on the blood of bats [5,6] as temporary parasites. Moreover, the population size of *S. wulingensis* is significantly correlated with the size of bat populations [9]. According to our field observation using infrared cameras in the study area where the species of *Sinospelaeobdella* were captured, no organisms were detected on the ceiling and vertical walls in the caves except bats. Therefore, we also predicted *Sinospelaeobdella* as the ectoparasite of bats.

In this study, we investigated a new species of *Sinospelaeobdella* found near Ganzhou City, Jiangxi Province, China, classified its taxonomic status within Haemadipsidae and refined the family’s phylogenetic framework. Our results provide basic data for the subsequent construction of a morphology-based phylogenetic tree and the study of the relationships among *Sinospelaeobdella* species, enhance the documented diversity of the recently established genus and provide scientific guidance for the determination and conservation of *Sinospelaeobdella*.

## 2. Materials and Methods

### 2.1. Sampling

**Figure 1:**
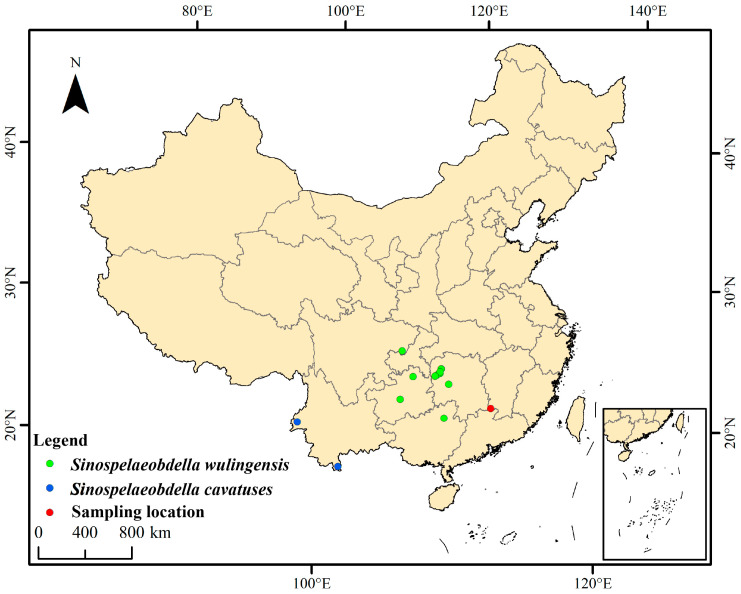
Distribution map of *Sinospelaeobdella* species currently discovered in China. The red point represents the sampling location of the novel species in the present study. The green and blue points represent the locations of *S. wulingensis* and *S. cavatuses* documented in the previous studies [8,9,10,11].

Specimens of land leeches were collected in dark areas of a natural cave near Niedu city, Jiangxi Province, China, located at 25°28′32.69′′ N, 114°7′38.96′′ E, 464 m above sea level (Figure 1). The location comprises a karstic cave without direct sunlight. The cave average temperature is 21.4 °C, and the relative humidity was 89.7% when we collected specimens.

All specimens were collected manually by Zhang Chen and Gu Hao. Specimens for morphological studies (JCS1~JXCS4) were collected in 2023, fixed, and preserved in 75% alcohol; specimens for molecular studies (JXCS6, JXCS7) were collected in 2022 and preserved in 100% ethanol.

### 2.2. Morphological Analysis

The specimens were collected, morphologically described and preserved following the established methodologies documented in the previous studies on *S. wulingensis* and *S. cavatuses* [6,8,9]. One holotype (JXCS1) and three paratypes (JXCS2~JXCS4) were collected. Voucher specimens and a research collection were deposited at the Jilin Provincial Key Laboratory of Animal Resource Conservation and Utilization (Northeast Normal University, Changchun, China).

Physical measurements and images were acquired using software (NIS-Element v4.2.0) mounted on a stereo zoom microscope (Nikon DS-Fi3, Nikon Corporation, Tokyo, Japan). Images were saved as TIF files, and editing and plate layout were performed in Adobe Photoshop.

### 2.3. Phylogenetic Analysis

**PCR and DNA sequencing.** We sequenced two individuals of the new species. A piece of the caudal sucker was removed and placed in 100% ethanol. The whole DNA extraction was performed using the Ezup Column Animal Genomic DNA Purification Kit (Sangon Biotech, Shanghai, China). Success of DNA extraction was confirmed using a 1% agarose gel electrophoresis test, and eluded genomic DNA was stored at −20 °C for later use.

The COI gene sequence was amplified using the universal primers LCO1490/HCO2198 [6,12] synthesized by Sangon Biotech. The total PCR-reaction volume was 25 µL, containing 1.0 µL template DNA, 2.5 µL 10 × Taq Buffer (with MgCl_2_), 1.0 µL 10 µmol/L of each primer, 1.0 µL 10 mmol/L dNTP, 0.2 μL 5 U/μL Taq DNA polymerase and 18 μL ddH_2_O. The PCR conditions were pre-denaturation at 95 °C for 5 min, 10 cycles with 94 °C denaturing for 30 s, annealing at 63 °C for 30 s (reduce 0.5 °C per cycle), extension at 72 °C for 30 s; 30 cycles with 95 °C denaturing for 30 s, annealing at 58 °C for 30 s, extension at 72 °C for 30 s, and a final extension at 72 °C for 10 min. The PCR products were sent to Sangon Biotech for purification and sequencing using the Sanger method on ABI 3730 (ABI, Los Angeles, CA, USA).

**Taxa sampling and multiple sequence alignment.** The sequencing results were compared and confirmed by Blast on NCBI. All new sequences were deposited in the Genbank database. Additional sequences were retrieved from GenBank, including known species of *Sinospelaeobdella* and several species of *Chtonobdella* and *Tritetrabdella.* The sequences of *Poecilobdella nanjingensis* were used as the outgroup. The final dataset consisted of 20 sequences (Table 1).

Multiple sequence alignment was performed using MUSCLE available from MEGA11 using default parameters. The aligned sequences were then trimmed to equal lengths using BioEdit 7.2.6.1 (Borland, Austin, TX, USA), and the final aligned length was 652 bp.

**Phylogenetic analysis.** After sequence alignment, the best nucleotide substitution model was calculated by MEGA11, and a Maximum Likelihood tree was constructed using the General Time Reversible (GTR) model with a gamma distribution of invariant sites (G + I). The number of bootstrap replications was set to 1000. The final tree was rooted with the outgroup (*Poecilobdella nanjingensis*) [13]. The constructed evolutionary tree was imaged by iTOL v6 (https://itol.embl.de/) accessed on 4 March 2024.

**Analysis of Genetic Distance.** The uncorrected pairwise genetic distance between COI sequences of the new species and of *S. wulingensis* and *S. cavatuses* was calculated using the Kimura 2-parameter model in MEGA 11.

## 3. Results

### 3.1. Taxonomy

**Family Haemadipsidae Blanchard, 1892**

**Genus *Sinospelaeobdella* Liu, Huang, and Liu gen. n.**

**Species *Sinospelaeobdella jiangxiensis* Li, Zhang, and Liu sp. n.**

**Etymology:** The specific name is derived from Jiangxi Province, the locality.

**Holotype:** Adult, intact, undissected. Chushui cave, Ganzhou city, Jiangxi, China, August 2023. Zhang Chen and Gu Hao, coordinates 25°28′32.69′′ N, 114°7′38.96′′ E, 464 m a.s.l. (JXCS1).

***Paratypes:*** Three adults, intact, undissected (JXCS2~JXCS4) Chushui cave, Ganzhou city, Jiangxi, China, August 2023. Zhang Chen and Gu Hao, coordinates 25°28′32.69′′ N, 114°7′38.96′′ E, 464 m a.s.l.

**Diagnosis**: Bloodsucking land leeches, approximately long conical, lacking pigment and stripes on the body surface, medium body size. Annuli with papillae and sensilla dorsally; papillae are apparent on the adult individual; papillae and sensilla more prominent on the reproductive individual. Five pairs of eyes arranged in a “U” shape, of which the second pair is the largest; the fourth and fifth pairs of eyes are spot-like and separated by three annuli. Testisacs in ten pairs, first pair of testes situated in XIV/XV. Crop ceca with 16 pairs; last annulus with anus on the top. Caudal sucker diameter greater than maximum body breadth dorsally, with three pairs of auricular projections and about 79–90 friction rays.

**Description:** Body length 25.50 ± 2.86 mm (n = 5), anterior (oral) sucker breadth 2.83 ± 0.42 mm, maximum body breadth 4.44 ± 0.76 mm, posterior (caudal) sucker diameter 5.05 ± 0.82 mm. The data were displayed by Mean ± SD.

**Figure 2:**
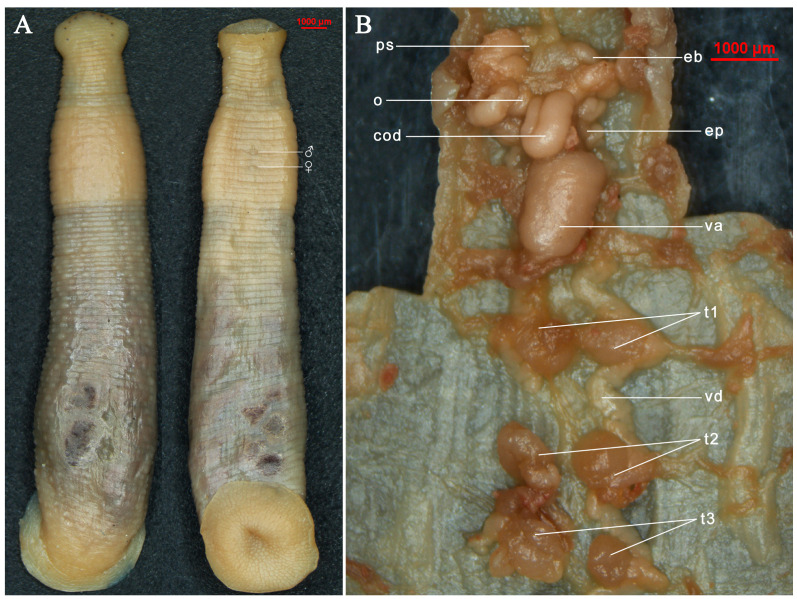
The morphological characteristics of *S. jiangxiensis* **sp. n.** (**A**): the dorsal and ventral views of holotype, the specimen (JXCS1) deposited in 75% alcohol; (**B**): the reproductive system of *S. jiangxiensis* **sp. n.** Abbreviations: ♂, male gonopore; ♀, female gonopore; cod, chief oviduct; eb, ejaculatory bulb; ep, epididymis; o, ovary; ps, penis sac; t1, first pair of testes; t2, second pair of testes; t3, third pair of testes; va, vagina sac; vd, vas deferens.

The body is medium in size and roughly long conical in shape, with slightly convex back and flattened venter (Figure 2A). The body is rather small in the anterior 1/4. The smallest posterior to the head and widest is at 3/4. The head and sucker are whitish and opaque; otherwise, they are brownish red due to the host blood ingested and appear creamy white or yellow when they are preserved in 75% alcohol (Figure 2A). The degree of feeding changes the size and shape of the body. The surface of the body lacks stripes or pigmentation dorsally and ventrally (Figure 2A). The annuli with papillae and sensilla dorsally is especially distinctive in reproductive individuals.

**Figure 3:**
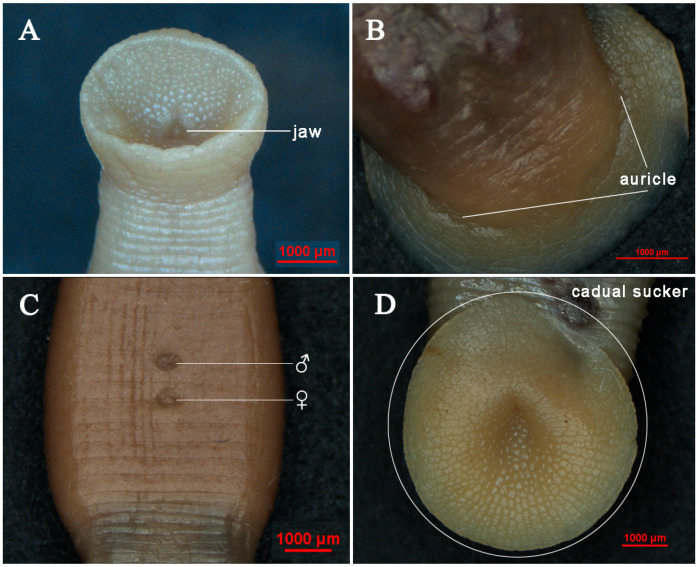
Partial external morphological characteristics of *S. jiangxiensis* **sp. n.** (**A**): jaw; (**B**): auricle; (**C**): position of gonopores: ♂: male gonopore, ♀: female gonopore; (**D**): cadual sucker (specimens preserved in 75% alcohol before photographing).

The entire body has 27 somites and 94 annuli in total, including somites I–IV 1-annulate; somite V 2-annulate; somites VI–VII 3-annulate; somite VIII 4-annulate; somites IX–XXII 5-annulate (b1 + b2 + a2 + b5 + b6); somite XXIII 3-annulate [b1 + b2 + (a2 + a3)]; somites XXIV 2-annulate; and somites XXV–XXVII singularly annulate. The number of complete somites is 5 at the edge of the anterior sucker lamelliform (Figure 2A). The anus is located at the middle of the last annulus (Figure 2A). The caudal sucker is nearly circular (Figure 3D), dorsally with three rows of auricular projection in annulus 92 to 94, where the first pair is large and conspicuous (Figure 3B). The last two pairs are small or barely noticeable (Figure 3B) with 79–90 friction rays (Figure 3D).

**Figure 4:**
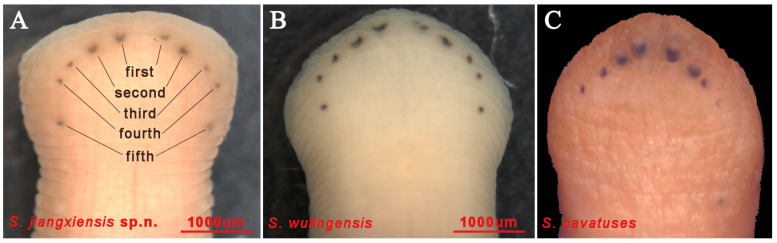
Ocular spot arrangement patterns in *Sinospelaeobdella*, illustrating interspecific variations in distribution and morphology. (**A**) *S. jiangxiensis* sp. n.; (**B**) *S. wulingensis*; (**C**) *S. cavatuses* [6]. *S. jiangxiensis* sp. n. and *S. wulingensis* both have the fourth and fifth pairs of eyes present, but the difference lies in that the fourth and fifth pairs of eyes of *S. jiangxiensis* sp. n. are separated by three annuli. In *S. cavatuses*, the first pair of eyes is the largest, whereas the fifth pair is very small or absent. Anterior sucker breadth: *S. jiangxiensis* sp. n. (**A**) ranges from 2.25–3.42 mm; *S. wulingensis* (**B**) ranges from 1.8–2.8 mm; and *S. cavatuses* (**C**) ranges from 2.6–3.0 mm [6].

The three jaws (trignathous) present as tall and pyramidal. The median jaw is larger than the lateral jaws. The teeth of the jaws are tiny and indistinct (Figure 3A). Salivary papillae are absent on all the jaws (Figure 3A). Five pairs of eyes arrange in a “U” shape, locating dorsally on the second, third, fourth, and sixth annulus, respectively, but the fifth pair locates on the ninth annulus (Figure 2A and Figure 4A). The second pair of eyes is much larger, while the fourth and fifth pairs of eyes are very small and spot-like, separated by three annuli (Figure 2A and Figure 4A).

Crop with 16 pairs of caecae, one pair in each of somites IX–XXII, one pair in somites XXIII–XXIV and XXV–XXVII. Smaller caeca in somites IX and X; in reproductive individuals, the caeca are smaller for somites IX–XII, probably due to compression of clitellum; caeca from somite XIV much larger; somites XX–XXII have the largest caeca; the last two pairs are smaller in size.

The new species is monoecious. The male reproductive organs are the first to mature and are subject to heterotrophic fertilization. The anterior half of the body has a clitellum that occupies seventeen annuli and thickens and becomes milky white during reproduction (Figure 2A and Figure 3C), distinguishing it from the rest of the body. Male gonopore in somite XII b2/a2; female gonopore in XII b5/b6; gonopores separated by two annuli (Figure 2A and Figure 3C). Male reproductive system with 10 pairs of spherical testisacs; the first pair of testes is located on the sides of the lower end of the XIV ganglion (Figure 2B). A vas deferens, which is an extension of the epididymis, connects the testes on each side. The epididymis consists of a thick tubular mass located on both sides of ganglia XII-XIII and connected anteriorly to the ejaculatory bulb (Figure 2B). The ejaculatory bulb, connected to the penis sac by the ejaculatory duct, is large and prominent. The penis sac is located on the underside of the XI ganglion, about three times the size of the ejaculatory bulb, and opens out through a short tube at the opening of the male gonopore in the XII ganglion (Figure 2B). A pair of ovaries lies to the left of ganglion XII, connected to the vagina sac by the oviduct and chief oviduct (Figure 2B). The vagina sac, located in ganglion XIII, is ellipsoidal and approximately 5–6 times larger than the penis sac (Figure 2B). It opens anteriorly through the thick vaginal duct from the female gonopore behind ganglion XII.

**Remarks:** The new species can be separated from *S. wulingensis* and *S. cavatuses* using the morphological characteristics listed in Table 2 and by COI sequence distance (Table 3). Specifically, *S. jiangxiensis* **sp. n.** differs from *S. wulingensis* in that the fourth and fifth pairs of eyes are separated by three annuli, and differs from *S. cavatuses* in that the second pair of eyes is the largest, and the fifth pair of eyes is present (Figure 4A–C). In addition, the shape and number of auricular projections can also be one of the diagnostic characteristics: *S. jiangxiensis* **sp. n.** has three pairs of auricular projections that are triangular, large, and distinct (Figure 3B), while in *S. cavatuses* the last two pairs of auricles are smaller or almost invisible. Additionally, *S. jiangxiensis* **sp. n.** can be distinguished from *S. wulingensis* and *S. cavatuses* by the number of friction rays (Figure 3D and Table 2), visibility of the papillae and sensilla dorsally (Figure 2A), the situation of the first pair of testes (Figure 2B), and measurements of the body size and other body parts.

**Figure 5:**
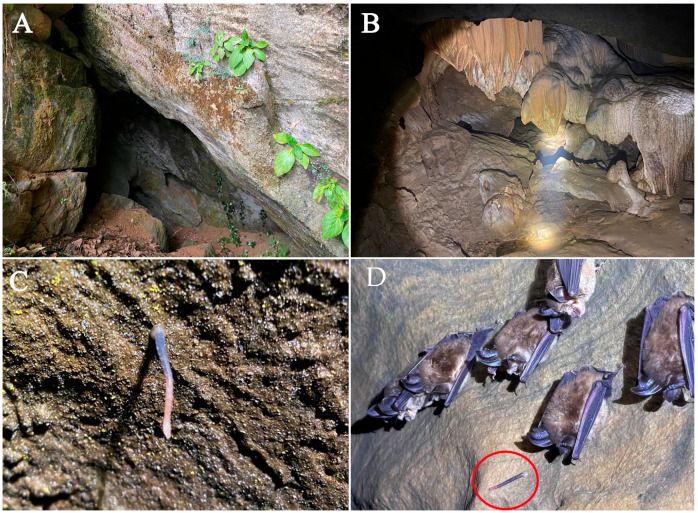
Habitat and collection location of *S. jiangxiensis* **sp. n.** (**A**): The entrance of the cave where the new species was captured; (**B**): The habitat of *S. jiangxiensis* **sp. n.** in the cave; (**C**): One individual of *S. jiangxiensis* **sp. n.** living on the ceiling of the cave; (**D**): Leech living close to the bats on the sidewall of cave.

**Figure 6:**
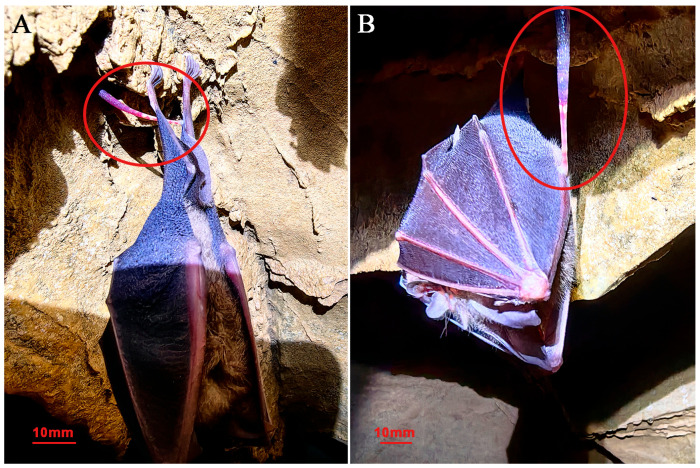
*S. jiangxiensis* **sp. n.** sucking blood from *Rhinolophus sp.* (**A**): sucking on the hindfeet; (**B**): sucking on the wing.

**Biology:** The new species lives on the roofs and sidewalls of wet karstic caves (Figure 5B–D), a stable microhabitat with an average temperature of 21.4 °C and relative humidity of 89.7%. The new species is monoecious and allogeneic. Reproductive individuals have large body size and dorsum papillae (Figure 2A). The newly discovered species were observed feeding on bat blood in natural habitats (Figure 6A,B), but the species exhibits stereotyped locomotor response characterized by the geometric postural shifts to the light stimuli or to human presence.

**Distribution:** The new species was captured in the caves of Ganzhou City in southwestern Jiangxi Province, China, where the dominant landscape is Karst, characterized by extensive solutional cave development.

### 3.2. Phylogenetic Analysis and Genetic Distance Comparison

**Figure 7:**
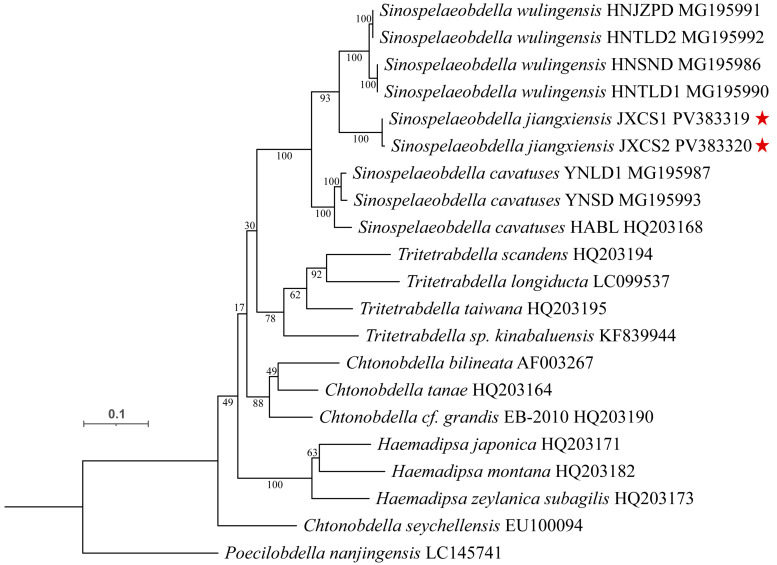
Phylogenetic relationship of different genera among the family Haemadipsidae. The maximum likelihood tree was built. The bootstrap replications were set to 1000. The stars indicate novel findings discovered in this study.

**Phylogenetic analysis:** A maximum likelihood tree was constructed based on the COI gene. *Poecilobdella nanjingensis* was selected as the outgroup. The results of the phylogenetic tree construction showed that the four genera of family Haemadipsidae were clustered into one clade. *S. jiangxiensis* **sp. n.** and *S. wulingensis* formed a sister group and further clustered as one clade with *S. cavatuses* (Figure 7).

**Genetic distance comparison:** The length of COI gene partial sequence was 679 bp, and the average contents of A, T, G, and C bases were 30.53, 12.39, 14.01, and 43.07%, respectively. The COI uncorrected mean Kimura 2-parameter genetic distance between the *S. jiangxiensis* **sp. n.** and *S. wulingensis* was 9.229%, and between *S. jiangxiensis* **sp. n.** and *S. cavatuses* was 10.995% (Table 3).

## 4. Discussion

Combined with the morphological characteristics and molecular evolution analysis, *S. jiangxiensis* **sp. n.** is regarded as a new species in the family Haemadipsidae, genus *Sinospelaeobdella*. *S. jiangxiensis* **sp. n.** possesses a ‘haemadipsine’ segmentation pattern, an ocular arch, and muscular tripartite jaws (Figure 2A and Figure 4) [2]. The new species can be separated from others in the genus by the number of annuli separating gonopores and the pigment and stripes on surface of body, and can be separated from other species of genus *Sinospelaeobdella* by the number and shape of auricle and eyes, the measurement of the body length and other body parts and the number of caeca and friction rays [6,8]. In addition, a lack of body pigmentation exists in the species of *S. jiangxiensis* sp. n., which is similar to the other two species of genus *Sinospelaeobdella*, but the feature is not observed in other genus of Haemadipsidae. The lack of body pigmentation might be due to the cave-dwelling dark life of *Sinospelaeobdella* [6,8,9]. The large variation in body size of the new species may be due to the differences between the foraging interval time as well as the degree of feeding of different individuals. The body size variation depending on feeding is common in other species of Haemadipsidae. Species of Haemadipsidae inhabiting forest and grassland typically breed during the rainy season and host activity periods, corresponding to seasonal reproduction. Studies on species of *Sinospelaeobdella*, such as *S. wulingensis*, reveal that their larvae and the individuals in their reproductive period exist year-round, indicating continuous reproduction that is likely to be due to the stable environmental conditions within caves [8,9,10]. The novel species in the present study also inhabits in stable cave ecosystems, and the larvae were always observed year-round. Therefore, we predict that the species engages in continuous reproduction, but this still needs further investigation.

Currently, species identification of *Sinospelaeobdella* is primarily based on the COI gene [4,6,7]. Maximum likelihood analysis of COI sequences revealed that the three species of *Sinospelaeobdella* were clustered into one clade, with *S. cavatuses* diverging earliest. *S. jiangxiensis* **sp. n.** and *S. wulingensis* show closer genetic affinity. Uncorrected pairwise distance analysis revealed that the average genetic divergence distance between *S. jiangxiensis* **sp. n.**, *S. wulingensis* and *S. cavatuses* exceeds the intraspecific and intersubspecific thresholds between other species of Haemadipsidae [14,15]. The results of phylogenetic analysis also support the classification of this novel species.

Jiangxi Province is located in southern China on the south bank of the middle and lower reaches of the Yangtze River; the area has a humid subtropical climate that provides favorable conditions for the survival and reproduction of various organisms [16]. Karst landforms are also widely distributed in Jiangxi Province, nurturing many cave-dwelling species. The new species lives on the roofs and sidewalls of wet karstic caves close to the bat populations coexisting in the same area. In addition to the cave where the samples of the new species were captured, we also found *S. jiangxiensis* **sp. n.** in the neighboring caves, although we did not capture samples because of the small population size. The genus *Sinospelaeobdella* has evolved specialized traits, such as lack of body pigmentation, ocular spot degradation, and dietary specialization that allows them to acclimatize to the cave environment [6,8,9,11]. The novel species exhibits troglomorphic adaptations, including reduction in ocular complexity and integumentary pigmentation, coupled with chemoreceptive elaboration, compared to photic-dwelling terrestrial leech species. It is assumed that, like *S. wulingensis*, the migration of bats led to the distribution of cave leeches to other caves [6]. The new species is therefore likely to be distributed in surrounding caves in nearby provinces, such as Guangdong, Hunan, and Guangxi, due to their similar topography and cave environments [4,6,7].

Leeches exhibit opportunistic predation strategies. According to previous studies, the terrestrial species of leeches living in forests and grasslands primarily feed on the blood or body fluid of various vertebrates, so they are not sensorily specific for certain species of host. However, cave-dwelling leeches, such as *S. wulingensis*, *S. cavatuses*, and *Leiobdella jawarerensis*, have been tested to show that they are mainly sensory specific for bat skin, although the biological mechanisms still need to be identified [6,8,17]. Bats roost or hibernate in caves, spending long periods perched on cave roofs or sidewalls, and have broad patagia and thin skins, presenting a resource for blood-sucking cave leeches. It has been documented that *S. cavatuses* is insensitive to human skin, but the leeches attach themselves to the patagia as soon as they encounter a bat and begin to suck blood, even if blood has already been stored in the crop ceca [8]. Analysis of the blood meal iDNA of *S. wulingensis* revealed that the species is monophagous, feeding only on the blood of bats, and that there was no evidence of feeding on animal taxa other than Chiroptera [5]. *Leiobdella jawarerensis* has also been found feeding on *Miniopterus tristris*, and laboratory experiments show that they will feed on young mice covered with fur [17]. Combinoing the morphological characteristics and the living environment of *S. jiangxiensis* **sp. n.**, the new species is predicted to feed mainly on the blood of bats.

Bats have been confirmed to carry and transmit viruses due to their large population size, intensive habitation behavior, and their ability to fly, while having no apparent symptoms of disease. More than 30 families and 54 genera of viruses have been identified in bats, including a wide range of zoonotic viruses that can be transmitted horizontally or vertically [18,19,20,21]. Bat-associated viruses could be transmitted mechanically or biologically by leeches of the genus *Sinospelaeobdella*. In addition, the leech could transmit mammalian trypanosome infection [17]. According to the prediction of the diet of *S. jiangxiensis* **sp. n.**, the dispersal of bats may enhance the dispersal of the species as a temporary parasite of bats. Therefore, the new species identified in the present study may be an additional potential virus transmission vector of bat pathogens, and the relationships between the cave leeches, the hosts, and pathogens and parasites should be addressed in future studies.

In conclusion, a new species of *Sinospelaeobdella* that adds to the extant taxa of Haemadipsidae was described. The results provide information for determining the phylogenetic status and evolution of Haemadipsidae. Our findings broaden the scope for studying diverse species in terms of the distribution and dispersal of *Sinospelaeobdella*.

## Figures and Tables

**Table 1:** COI sequences included in analysis of phylogenetic relationship between known populations of Haemadipsidae species. The bolded words are the genus name and outgroup.

Species	Sample No.	Location	GenBank Acc	Reference
***Sinospelaeobdella***				
*S. jiangxiensis* **sp. n.**	JXCS6	Jiangxi, China	PV383319	This study
*S. jiangxiensis* **sp. n.**	JXCS7	Jiangxi, China	PV383320	This study
*S. wulingensis*	HNSND	Hunan, China	MG195986	Huang et al. 2019 [6]
*S. wulingensis*	HNTLD1	Hunan, China	MG195990	Huang et al. 2019 [6]
*S. wulingensis*	HNJZPD	Hunan, China	MG195991	Huang et al. 2019 [6]
*S. wulingensis*	HNTLD2	Hunan, China	MG195992	Huang et al. 2019 [6]
*S. cavatuses*	HABL	Luang Namtha, Laos	HQ203168	Borda et al. 2010 [2]
*S. cavatuses*	YNLD1	Yunnan, China	MG195987	Huang et al. 2019 [6]
*S. cavatuses*	YNSD	Yunnan, China	MG195993	Huang et al. 2019 [6]
***Chtonobdella***				
*C. tanae*	AU79	Australia	HQ203164	Borda et al. 2010 [2]
*C.* cf. *grandis*	EB-2010	Australia (Tasmania)	HQ203190	Borda et al. 2010 [2]
*C. bilineata*		Australia (New South Wales)	AF003267	Siddall 1998
***Tritetrabdella***				
*T. taiwana*	TICH	Guanxi, China	HQ203195	Borda et al. 2010 [2]
*T. scandens*	TI49	Thailand	HQ203194	Borda et al. 2010 [2]
*T. longiducta*	VNMN04733	Vietnam	LC099537	Nakano et al. 2016 [13]
*T. kinabaluensi inobongensis*	SP13380	Malaysia (Sabah)	KF839944	Kappes 2013 [14]
***Haemadipsa***				
*H. japonica*	HAJA	Japan	HQ203171	Borda et al. 2010 [2]
*H. zeylanica subagilis*	HAN04	Thailand	HQ203173	Borda et al. 2010 [2]
*H. montana*	HZKI	Nepal	HQ203182	Borda et al. 2010 [2]
**Outgroup**				
*Poecilobdella nanjingensis*		Taiwan, China	LC145741	Nakano et al. 2016 [13]

**Table 2:** Comparison of morphology between *S. jiangxiensis* sp. n., *S. wulingensis* and *S. cavatuses*.

Items	*S. cavatuses* [8] n = 13	*S. wulingensis* [4,6,7] n = 25	*S. jiangxiensis* sp. n. n = 5
Body length (mm)	31~44	15~28	22.66~28.89
Maximum body breadth (mm)	6.0~8.0	3.5~5.4	3.75~5.67
Anterior sucker breadth (mm)	2.6~3.0	1.8~2.8	2.25~3.42
Posterior sucker diameter (mm)	6.0~7.5	4.1~6.7	3.91~5.95
Jaws	3	3	3
Complete somites	5 annuli	5 annuli	5 annuli
Largest eyes	first pair	second pair	second pair
Fifth pair eyes	small or absent	present	present
The dorsum papillae	large and obvious	small	large and obvious
Caeca pairs	10	14	16
Testisac pairs	10	10	10
Friction rays	78	78	79~90

**Table 3:** The uncorrected pairwise genetic distance between COI sequences from samples representing *S. jiangxiensis* sp. n., *S. wulingensis* and *S. cavatuses*.

	Genbank no.									
*S. jiangxiensis*	PV383319									
	PV383320	0.0030								
*S. wulingensis*	MG195991	0.0893	0.0892							
	MG195990	0.0943	0.0943	0.0162						
	MG195992	0.0893	0.0892	0.0000	0.0162					
	MG195986	0.0943	0.0943	0.0177	0.0015	0.0177				
	MG195994	0.0943	0.0943	0.0177	0.0015	0.0177	0.0000			
*S. cavatuses*	MG195987	0.1094	0.1094	0.1080	0.1063	0.1080	0.1046	0.1046		
	HQ203168	0.1094	0.1094	0.1097	0.1080	0.1097	0.1097	0.1097	0.0404	
	MG195993	0.1111	0.1111	0.1063	0.1046	0.1063	0.1063	0.1063	0.0162	0.0389

## Data Availability

The original data presented in the study are openly available in NCBI: PV383319, PV383320. Zoobank LSID: urn:lsid:zoobank.org:pub:D9B14C5B-F6B2-4AAE-A32B-172F6 128A07D. The description and nomenclature of this species follow the International Code of Zoological Nomenclature (ICZN, online version accessed on 7 April 2025).
